# Application of a Machine Learning-Based Classification Approach for Developing Host Protein Diagnostic Models for Infectious Disease

**DOI:** 10.3390/diagnostics14121290

**Published:** 2024-06-18

**Authors:** Thomas F. Scherr, Christina E. Douglas, Kurt E. Schaecher, Randal J. Schoepp, Keersten M. Ricks, Charles J. Shoemaker

**Affiliations:** 1 Atticus Labs, Baltimore, MD 21212, USA; tom@atticuslabs.net; 2 Diagnostic Systems Division, U.S. Army Medical Research Institute of Infectious Diseases, Fort Detrick, MD 21702, USAschoepphome@verizon.net (R.J.S.); keersten.m.ricks.civ@health.mil (K.M.R.); 3 Virology Division, U.S. Army Medical Research Institute of Infectious Diseases, Fort Detrick, MD 21702, USA

**Keywords:** machine learning, biomarker, host, diagnostic, protein, classification, infectious disease

## Abstract

In recent years, infectious disease diagnosis has increasingly turned to host-centered approaches as a complement to pathogen-directed ones. The former, however, typically requires the interpretation of complex multiple biomarker datasets to arrive at an informative diagnostic outcome. This report describes a machine learning (ML)-based classification workflow that is intended as a template for researchers seeking to apply ML approaches for developing host-based infectious disease biomarker classifiers. As an example, we built a classification model that could accurately distinguish between three disease etiology classes: bacterial, viral, and normal in human sera using host protein biomarkers of known diagnostic utility. After collecting protein data from known disease samples, we trained a series of increasingly complex Auto-ML models until arriving at an optimized classifier that could differentiate viral, bacterial, and non-disease samples. Even when limited to a relatively small training set size, the model had robust diagnostic characteristics and performed well when faced with a blinded sample set. We present here a flexible approach for applying an Auto-ML-based workflow for the identification of host biomarker classifiers with diagnostic utility for infectious disease, and which can readily be adapted for multiple biomarker classes and disease states.

## 1. Introduction

Infectious diseases represent a significant burden on human health, particularly in austere or resource-limited settings. Despite advances in medical diagnostic capability, the persistence of this issue, particularly in areas of the world with endemic and emerging diseases, poses an increasing challenge. The majority of infectious disease diagnostic assays are pathogen-focused tests that support accurate clinical decision-making; the most common types being culture, polymerase-chain reaction (PCR), and serology. Pathogen-targeted assays, however, can only detect what they are designed against and are blind to novel or unexpected threats, as was seen for both the 2009 H1N1 influenza and SARS-CoV-2 pandemics [1,2,3,4]. Furthermore, it can take considerably longer to develop sensitive and specific tests for an emerging pathogen. Even after the development of pathogen-specific diagnostic tests, these assays are sometimes limited in sensitivity until the pathogen has reached a critical bioburden at which point it can be detected in the sample matrix (e.g., viral load in whole blood), potentially delaying any effective clinical intervention. In consideration of this, applications exist where a rapid, flexible identification strategy that is not dependent on pathogen culture or targeted assays would be ideal.

An alternative to pathogen-specific identification lies in disease complementary approaches to infectious disease that instead focus on host response [5,6]. Measurement of host response to infection can encompass numerous biomarker classes, including soluble proteins, cytological profiles, nucleic acids, and assorted metabolites. Regardless of the specific biomarkers used, this type of diagnostic approach is pathogen agnostic; it does not require a priori knowledge of the specific pathogen to be viable, instead relying on perturbations to host biomarker homeostasis [7]. Detection of host biomarker changes in response to infection can also be quite rapid, sometimes even preceding direct detection of the pathogen itself [8,9,10]. As most of these soluble biomarkers exist at detectable endogenous levels in healthy specimens, a threshold cutoff value typically segments diagnostic outcomes based on differences from the nominal healthy baseline. For example, a research group recently identified a panel of five host proteins present in plasma that could accurately predict a patient’s Tuberculosis status up to 2 years prior to formal diagnosis based on detection of Mycobacterium tuberculosis in patient biofluids [11]. In such a scenario, clinical access to prognostic and/or diagnostic tools that are independent of direct pathogen detection represents a significant value. Successful deployment of such clinical diagnostic assays holds significant benefits including the ability to rapidly repurpose existing assays for novel threats and to make timely and effective clinical interventions. In the case of bacterial vs. viral etiology identification, this could inform patient care with decisions like antibiotic use, quarantine from other patients, and general patient risk stratification [12]. To realize this potential, considerable obstacles must be overcome, most significantly the ability to correctly interpret multiparametric data. Due to patient-to-patient disease variance, reliance on a single host biomarker for a disease diagnostic may have poor diagnostic value [13]. This is compounded by the fact that host proteins can be impacted by a range of factors, microbial as well as non-microbial influences including age, diet, obesity, stress, and individual genetics [14]. To address this relative lack of non-specificity at the single biomarker level, an alternate approach involves relying on multiplexed pools of host biomarker analytes to increase both assay diagnostic performance and robustness. Introducing this complexity into an assay, however, poses challenges to the definition of an exact classifier for determining the diagnostic outcome.

Machine learning (ML) is a subfield of artificial intelligence that has rapidly gained traction in recent years in several areas, including biology [15,16,17]. ML approaches have recently been utilized to untangle complex, interdependent features to elucidate new biomedical insights, particularly in the cancer and infectious disease fields [18,19,20,21]. The sophisticated algorithms employed have demonstrated the capability to discern subtle differences and detect correlations that might elude traditional statistical methods or human analysis; this is especially true with multivariate datasets. For the classification of host response biomarkers, the identification of an infection’s etiology from a series of biomarker concentrations derived from a set of specimens is well-posed as a supervised ML classification problem. It has clearly defined numerical inputs (i.e., the biomarkers’ measured laboratory values) associated with a categorical output (i.e., bacterial, viral, no infection, or indeterminate). ML is uniquely suited to handle the inherent complexity of host immune response variability, whereas traditional approaches often struggle to account for this complexity, leading to delayed or inaccurate diagnoses. ML models excel in this context by their ability to process and analyze vast datasets, identifying patterns and correlations that are not immediately apparent.

An application where considerable work has been conducted in the use of host-based biomarkers for infectious disease diagnosis is in bacterial versus viral disease differentiation [22]. Rapid sample-to-answer solutions for this problem at the point of care enable better antibiotic stewardship, improve decisions on patient isolation, and inform further pathogen-specific diagnostics downstream (e.g., requesting a bacterial or viral-specific PCR panel). Procalcitonin (PCT) and C-reactive protein (CRP) have been used for many years as diagnostics for bacterial sepsis [23]. Over the past decade, multiple groups have also published different host-based signatures for this purpose. Most of these are based on the detection of protein biomarkers such as CRP, interferon-γ-induced protein-10 (IP-10), and TNF-related apoptosis-induced ligand (TRAIL) [24,25,26,27,28,29]. As mentioned above, however, identifying the correct combinations and “weights” of different biomarkers in a classifier can be difficult. The interpretation of such complex datasets and the elucidation of useful biomarker classifiers for identifying disease etiology is fertile ground for some of the tools offered by recent advances in artificial intelligence.

As agnostic, host-centered approaches to disease identification become more prevalent, machine learning can be leveraged to identify multi-parametric biomarker classifiers with robust predictive power. To this end, we applied an ML-guided approach to the challenge of differentiating viral, bacterial, and normal clinical samples as a case study; similar publications have been released for the application of ML methodologies in other avenues of biomedical research [30,31]. The primary intent of this manuscript is to present an effective template workflow for designing ML pipelines for building multiplexed, host-based infectious disease classifiers. A secondary objective of this work is to demonstrate the capabilities of automated machine learning (Auto-ML) platforms, emphasizing their potential utility for individuals without extensive expertise in machine learning to rapidly develop high-performing models for infectious disease diagnostics.

## 2. Materials and Methods

### 2.1. Institutional Review Board Statement

All data and human participants research were previously de-identified and given “research not involving human participants” determinations by the U.S. Army Medical Research Institute of Infectious Diseases (USAMRIID) Office of Human Use and Ethics; log numbers FY17-08 and FY17-31.

### 2.2. Human Clinical Samples

The initial round of wet lab sample testing consisted of de-identified human clinical serum samples selected from the United States Army Medical Research Institute of Infectious Diseases (USAMRIID, Ft. Detrick, MD, USA), BioIVT Inc. (Westbury, NY, USA), Walter Reed National Military Medical Center (WRNMMC, Silver Spring, MD, USA), and Theratest laboratories (Lombard, IL, USA). Healthy donor sera were sourced from all four entities. WRNMMC provided 76 samples positive for *Escherichia coli*, *Enterococcus faecalis*, *Enterobacter asburiae*, *Citrobacter koseri*, *Entercoccus* sp., *Klebsiella pneumonaie*, *Pantoea agglomerans*, *Staphylococcus* sp., *Staphylococcus aureus*, Cytomegalovirus, Epstein–Barr Virus, Hepatitis B and Hepatits C. All WRNMMC human clinical samples were residual material collected in the course of normal clinical patient management and treatment. BioIVT provided 57 samples positive for *Borrelia burgdorferi*, *Chlamydia trachomatis*, *Neisseria gonorrhoeae*, *Treponema pallidum*, Hepatitis A, Hepatitis B, Hepatitis C, Influenza A, Influenza B, Influenza sp. and Dengue virus. For the blinded evaluation, USAMRIID provided 16 human serum samples positive for Chikungunya virus that were sourced from a disease outbreak in Puerto Rico that occurred in 2014 [32]. Work with Chikungunya samples was undertaken by trained personnel in a BSL-3 containment suite. All other work was completed at BSL-2 with appropriate safety precautions. For infectious samples, we focused on acute, active disease samples; infectious disease etiology was ascribed to samples based on a combination of direct pathogen detection (i.e., culture or PCR) and other clinical parameters.

### 2.3. Host Protein Immunoassays

All human clinical serum samples were processed using the Luminex MAGPIX^®^ instrument (DiaSorin Inc., Saluggia, Italy) and commercially available assays from R&D Systems (Minneapolis, MN, USA) and CloudClone (Katy, TX, USA). The CloudClone kit is a single-plex Immunoassay that detects the MxA protein. All other proteins were detected by R&D Biosystems Immunoassays including a 1-plex kit (C-Reactive Protein/CRP), a 2-plex kit (Ferritin & Lipocalin-2/NGAL) and a 9-plex kit (TNF-alpha, IL-6, IL-8/CXCL8, CXCL10/IP-10/CRG-2, IL-10, TRAIL/TNFSF10, Procalcitonin/PCT, IL-2 & IL-4). Samples were diluted in the provided assay diluent for use in each assay based on manufacturer recommendations, except for the MxA assay, which recommended using 100 µL of each sample. A 2-fold dilution was used for the MxA assay due to sample availability. Manufacturer protocols were followed for all R&D Biosystems Immunoassays. CloudClone protocols were followed for the MxA assay, except samples were incubated at room temperature and humidity. Analyte concentrations were extrapolated based on standard curves provided with each kit. Data were extracted as CSV files, which were then used for downstream analysis.

### 2.4. Analysis Hardware

All data visualization and machine learning model development was performed on a dedicated server with the following specifications: 3.2 GHZ 8-Core Intel Xeon W processor, Radeon Pro Vega 56 8 GB graphics, and 32 GB 2666 MHz DDR4 memory.

### 2.5. Analysis Software

Analysis was performed using the LabAble^®^ data analysis package (v1.1.3, Atticus Labs, Baltimore, MD, USA), with additional custom software modification. The software package is written in Python (v3.9.16) [33], and uses open-source packages for data loading, filtering, manipulations (Pandas v1.5.3) [34], data visualization (Seaborn v0.12.2 [35], Matplotlib v3.6.3) [36], and machine learning model building, training, and performance analysis (SciKitLearn v1.2.2 [37], mljar-supervised v0.11.5) [38,39]. All packages used in this work are freely available and have permissive licenses (e.g., MIT, BSD, Python Software Foundation). Data were cleaned for missing, incomplete, or incompatible values prior to usage; this included the conversion of undetectable biomarker values to the minimum of the detectable threshold of the specific assay. Exploratory data analysis (EDA) was performed to identify initial patterns, outliers, and gain a general understanding of the data. Prior to machine learning model training, target labels (i.e., infectious etiologies) were encoded with numerical values, and non-numeric columns in the dataframe pertaining to sample identification and source were dropped. The auto-ML library used in this study, mljar-supervised, automatically applies normalization to non-tree-based methods (e.g., linear, neural networks). The scaling method used was SciKitLearn’s StandardScaler, which removes the mean and scales to unit variance, creating a mostly normal distribution. Following a typical Auto-ML workflow, models of increasing complexity but decreasing interpretability were built: exploratory/preliminary models, performance models, and optimized models. At each stage, different model architectures were trained and evaluated, including the following: baseline models (always predicts the most common class from the training data), linear regression, decision trees, neural networks, Xgboost, Catboost, random forest, and an ensemble of these models. In earlier stage models, feature importance metrics were analyzed to inform any model or feature downselections. In later stages, more advanced hyperparameter tuning was performed to maximize performance.

### 2.6. Machine Learning Model Performance Metrics

Several common statistical metrics were used to evaluate the machine learning models, both training and diagnostic performance:

Sensitivity (recall, true positive rate): the proportion of true positives out of the total actual positive cases (TP + FN). It is calculated as Sensitivity = TP/(TP + FN).

Specificity (true negative rate): the proportion of true negatives out of the total actual negative cases (TN + FP). It is calculated as Specificity = TN/(TN + FP).

Positive Predictive Value (PPV) (precision): the proportion of true positives out of the total predicted positive cases (TP + FP). It is calculated as PPV = TP/(TP + FP).

Negative Predictive Value (NPV): the proportion of true negatives out of the total predicted negative cases (TN + FN). It is calculated as NPV = TN/(TN + FN).

Accuracy: the proportion of correct predictions (both true positives and true negatives) out of the total predictions (TP + TN + FP + FN). It is calculated as Accuracy = (TP + TN)/(TP + TN + FP + FN).

F1 score: a commonly used performance metric in machine learning that combines precision and recall into a single value. It is particularly useful when both precision and recall are equally important in a problem, and a balance between the two is desired. The F1 score is the harmonic mean of precision and recall, and is calculated using the following formula:

F1 Score = 2 × (Precision × Recall)/(Precision + Recall)

The F1 score ranges between 0 and 1, where a higher value indicates a better model performance. The F1 score is often used in binary classification problems, where there are only two classes: positive and negative. However, it can also be extended to multi-class problems using micro-averaging (each model prediction treated equally, regardless of the prediction’s class) or macro-averaging techniques (each class treated equally, regardless of the number of predictions in that class) when multiple classes are involved.

Logloss: short for logarithmic loss, also referred to as cross-entropy loss, is a common evaluation metric used in machine learning to measure how well a classification model is performing. Logloss measures how different the predicted probabilities of a model are from the actual outcomes. The lower the logloss value, the better the model is at predicting the correct outcome for each observation. Logloss is the method primarily utilized throughout this work to describe model performance during training. The multiclass logloss, which reduces to a binary logloss in the event of only two class labels, can be calculated with the following formula [37]:

$$L_{\log}\left(Y,\;P\right)=-\log\mathrm{Pr}\left(Y|P\right)=-\frac{1}{N}\overset{N-1}{\underset{i=0}{\sum }}\overset{K-1}{\underset{k=0}{\sum }}y_{i,k}\log p_{i,k}$$

The application of these metrics for binary classification problems is straightforward. For multi-class classification problems, where there are more than two target classes, these metrics can be calculated in different ways depending on the context and specific requirements of the problem. A common approach for multi-class problems, used in this work, is to treat each target class as the positive class and calculate the sensitivity for that class against the rest of the classes combined as the negative class. This is often referred to as “one-vs-rest” or “one vs. all” approach.

### 2.7. Additional Statistical Calculations

To evaluate the distribution of laboratory values for each biomarker across the different classes, we initially conducted Shapiro–Wilk and Levene’s tests to assess normality and equality of variances—conditions required for the application of ANOVA for determining significance. As these conditions were not met, we employed the Kruskal–Wallis test to evaluate significance across the three classes. If this test indicated significant differences, we proceeded with Dunn’s test, applying a Bonferroni correction for pairwise comparisons.

Similarly, to evaluate the distribution of the blinded viral specimens to the distributions of the viral training set, we initially conducted a Shapiro–Wilk test to assess the normality—required for the application of a *t*-Test. As either the blinded viral specimen distribution or the viral training set distribution was not normal, we used a Mann–Whitney U test with a Bonferroni correction to evaluate the significance across the two training sets for each biomarker.

The correlation ratio was used to evaluate how well the categorical variables (i.e., classes) explain the variance of the numerical variables (i.e., biomarker distributions). It was calculated as follows:

$$\eta^{2}=\frac{\sum_{x}n_{x}{\left({\bar{y}}_{x}-\bar{y}\right)}^{2}}{\sum_{i}{\left(y_{i}-\bar{y}\right)}^{2}}$$

Here, $\eta^{2}$ is the square of the correlation ratio, *x* is a particular class, $n_{x}$ is the number of observations in class *x*, ${\bar{y}}_{x}$ is the mean of the observations in class *x*, $\bar{y}$ is the mean of all observations across all classes, *i* is the index of a particular observation, and $y_{i}$ is the individual observation.

## 3. Results and Discussion

**Figure 1:**
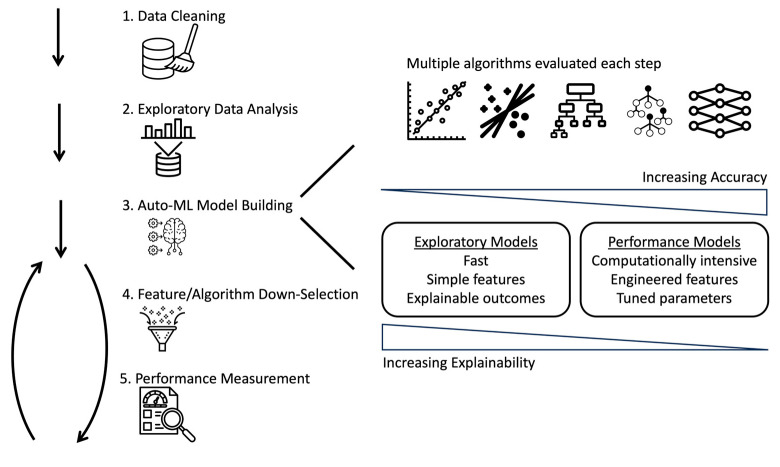
Flow chart describing the iterative steps to build an Auto-ML pipeline: data preprocessing, modeling training, and performance evaluation.

We employed an iterative, Auto-ML pipeline for building our infectious disease classifier model. An overview of this workflow is summarized in Figure 1. In this approach, multiple ML algorithms are trained and tested at each stage, model performance is analyzed, and, finally, features and ML algorithms are downselected. Exploratory models that are fast and have explainable outcomes are utilized at the outset, ultimately giving way to more computationally intensive and less explainable, but more accurate, performance models with optimized parameters.

### 3.1. Data Collection, Cleaning, and Exploratory Analysis

For this case study, we first assembled a collection comprised of human sera samples of known bacterial, viral, or normal etiology (Table 1). There were 221 valid samples for analysis included in the dataset. All samples had a target class, “infection identifier”, which was the label to be used for machine learning model training and testing. Of this target class, the dataset contains the following distribution across its classes: normal = 88, viral = 71, bacterial = 62. There is a slight class imbalance in this training set, so care was exercised when evaluating models to ensure that they were not biased towards the predominant “Normal” infection identifier. There were 23 clinical disease designations (14 bacterial and 9 viral) which were in turn segmented into bacterial and viral classes with infection, and 1 class for “None” (associated with healthy/normal specimens). Next, we subjected these samples to multiplexed immunoassays targeted against an array of host proteins that have been reported in the literature in recent years for infectious disease research and especially for bacterial/viral differentiation. These included MxA, C-Reactive Protein/CRP, Ferritin, Lipocalin-2/NGAL, TNF-alpha, IL-6, IL-8/CXCL8, CXCL10/IP-10, IL-10, TRAIL/TNFSF10, Procalcitonin/PCT, IL-2, and IL-4 [24,25,26,27,28,29,40]. With the exception of MxA, all of these biomarkers are secreted and have been detected in sera and plasma. As a predominantly intracellular biomarker, the interferon-inducible protein MxA is an exception; however, it has been reported in some studies as a biomarker of viral infection in whole blood in multiple studies [41,42]. We chose to include it in this panel based on preliminary evidence from our group suggesting it could be reliably detected in extracellular matrices like serum.

Following data collection, data were cleaned as follows. All numeric data were analyzed for NaNs (not-a-number values in numeric columns). This dataset comes from laboratory assays with minimum signal cutoff values, and when a specimen had an immeasurable amount of biomarker, the instrument output applies a minimum threshold value as a text string (e.g., “<0.09765625”, “<3.7037037037037”, “<6.25514403292181”, “<36.9958847736625”). The raw data contained values in this form, which must be converted to a numerical value for visualization and machine learning purposes. Two common solutions to this approach are as follows: (1) setting these values to zero, (2) setting these values to the minimum cutoff threshold value. Since each independent assay has a different scale, and these thresholds span multiple orders of magnitude, the latter approach was used: the result of any assay that was undetectable was set to the minimum threshold for that assay.

**Figure 2:**
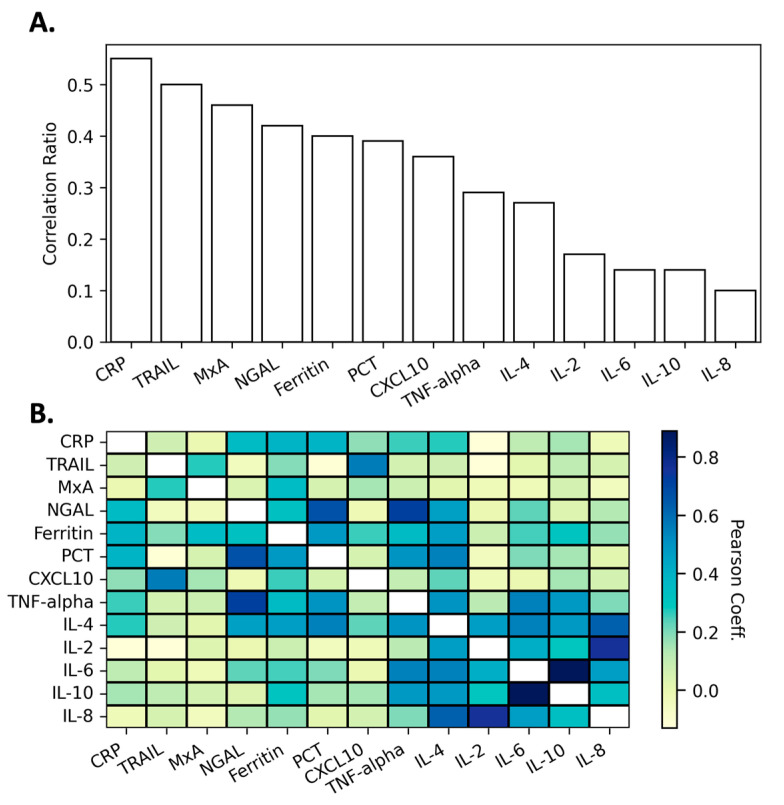
Exploratory biomarker data. (**A**) Single biomarker correlations with sample classifier status. (**B**) Heat map showing correlations between different biomarkers.

After data cleaning, we performed exploratory data analysis looking at individual biomarker distributions. Each of the 13 biomarkers of interest is represented in the dataset as a numeric value. It is helpful to first visualize the distributions of individual biomarkers (SI Figure S1, segregated by their target class “infection identifier”). Having carried this out, multiple preliminary conclusions were readily apparent: no single biomarker value is likely to be sufficiently discriminatory to be used as a classifier for all three infection identifiers, there are biomarkers that are highly correlated with a single target infection identifier, and it is likely that machine learning model performance will improve when the objective is to classify a single infection identifier rather than discriminate against all possible outcomes (e.g., bacterial vs. not-bacterial (viral + normal)). The distributions of protein biomarkers were noted to exhibit outliers for their given infection identifiers (39.8% of specimens with at least one biomarker outliers, SI Figure S2). Outliers were more frequent in bacterial and viral specimens than in normal sera, and they were not uniformly distributed across all biomarkers. When samples were found to have an outlier, it most often was observed as a single biomarker outlier (25.8% of specimens); 14.0% of specimens were observed to have more than one biomarker outlier (9.9% 2 outliers, 3.2% 3 outliers, 0.9% 4 outliers). We elected not to remove outliers from the training data set, based on the recognition that outliers could represent biologically significant variations in human populations, possibly reflecting severe disease states. These extreme values might carry critical biological insights and excluding them could potentially omit valuable information. As there is a target variable of interest (i.e., infection identifier), it is logical to calculate the correlation ratios between individual biomarker data and this categorical variable. Examining each of these biomarkers in isolation, we performed correlation analysis evaluating the ability of each individual host protein to correlate to its categorical disease state (Figure 2A). We also examined the correlative interactions between the individual analytes to assess biomarkers that were likely to behave similarly (Figure 2B), as this may impact biomarker downselection. Interestingly, we observed several instances of sets of proteins (e.g., TRAIL/CXCL10 and NGAL/PCT/TNF-alpha) that responded to infection in a similar manner. This reinforces findings from the field that describe the complex network effects and/or redundancy that are observed in biomarkers associated with host pathogen-response pathways [40,43].

Prior to machine learning model building, the following steps were taken to further pre-process the dataset: (1) target labels (“infection identifier”) were encoded with numerical values (0: bacterial, 1: normal, 2: viral) and (2) all non-numeric columns were dropped from the dataset (“Sample”, “Source”, “Organism”).

### 3.2. Impact of Auto-ML Modeling on Model Performance Metrics

**Figure 3:**
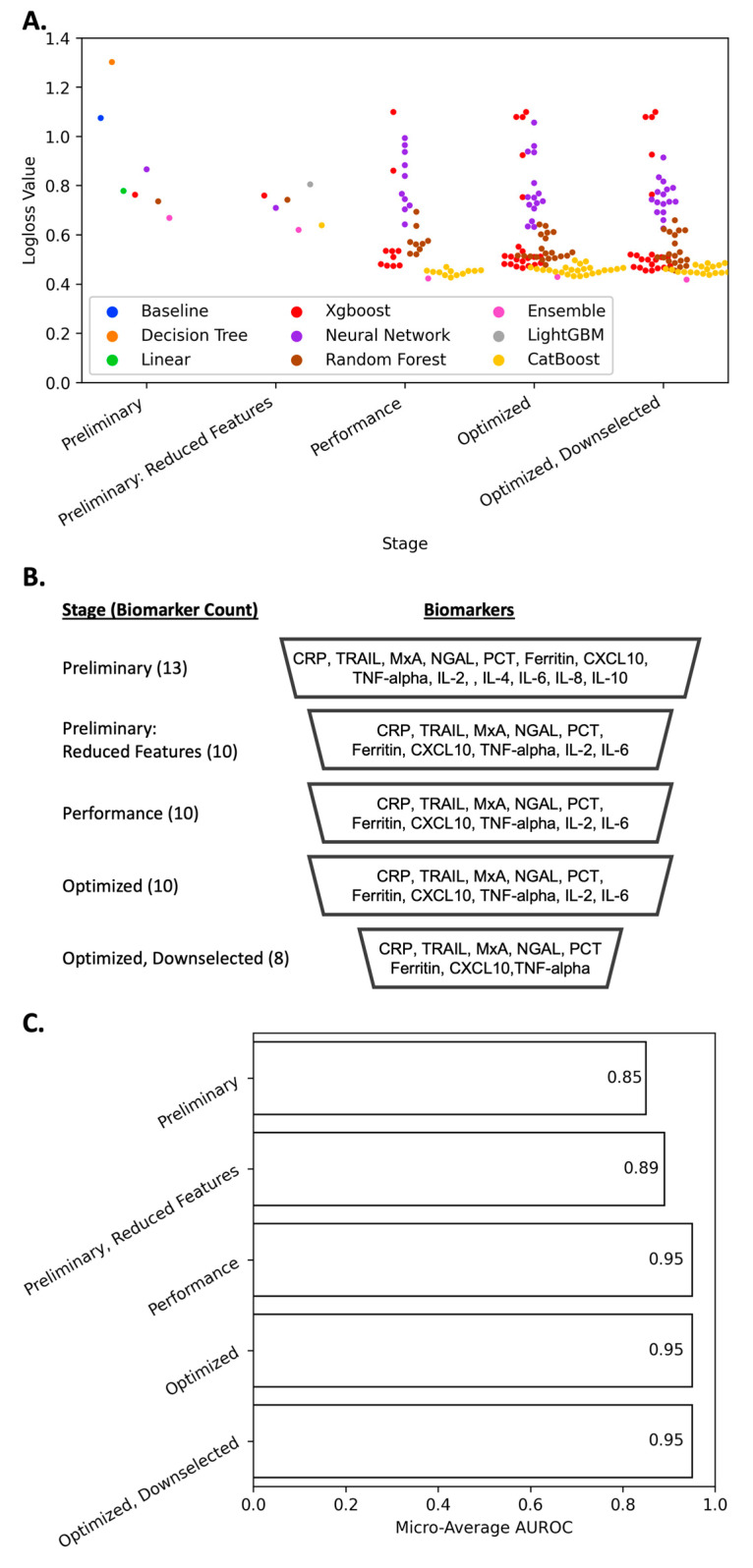
(**A**). Performance improvement of different ML models over the course of iterative Auto-ML process. Lower logloss scores convey improved model performance metrics relative to true classifier identity. (**B**). Biomarker features used at each stage of machine learning model development. (**C**). The micro-average of the area under the ROC curve (AUROC) for the best performing model at each stage. For each model, a ROC curve is made for each class individually, and then the micro-average is an aggregate measurement that takes the average across all classes, thus giving each class equal importance.

Preliminary modeling quickly evaluates several machine learning algorithms with default hyperparameters and can be useful at the outset to evaluate the following: (1) whether machine learning is an appropriate tool, (2) which features may be the most important, and (3) which models should receive the most focus for rigorous tuning. Preliminary modeling has the highest level of “explainability”, as the goal of this stage is still to gain an understanding. In this preliminary modeling, the dataset is split into 75%/25% train/test sets, and 7 common ML models were evaluated: Baseline (a crude model that returns the most frequent class as a prediction), Decision Tree, Neural Network, Linear, Xgboost, Random Forest, and an Ensemble model of the other models combined. Comparing models (by computing the percentage difference of the logloss metric used for model evaluation) to the Baseline is a useful means of evaluating the need for machine learning approaches. As is shown in Figure 3A, with the lone exception being the Decision Tree algorithm, all algorithms outperformed the Baseline model. The best performing model (Ensemble) showed a 37.7% improvement over the Baseline model. An empirical heuristic that can be applied here would be that if model performance is less than 5% of an improvement over the Baseline, then the data would appear to be random and the use of machine learning modeling approaches should be reconsidered. Even applying this heuristic cautiously, this preliminary modeling would suggest that the usage of machine learning is justified and that the dataset does not appear to be random/noisy data.

Given that connected immune and inflammatory pathways regulate responses to exogenous pathogens, and quantities of biomarkers within these pathways are used as features in the ML models, feature importance metrics can provide further insight and assist in the refinement of the ML pipeline. SHAP (Shapley Additive Explanations) [44] feature importance values for the random forest model in the preliminary model stage (SI Figure S3), the best performing model at this stage, mostly show agreement with the infection identifier correlation ratios (Figure 2A). More useful at this stage, there is agreement on the least important features, and we chose to remove IL-8, IL-10, and IL-4 as features. While having low feature importance scores, Ferritin and TNF-alpha were not downselected due to their higher infection identifier correlation ratios.

**Figure 4:**
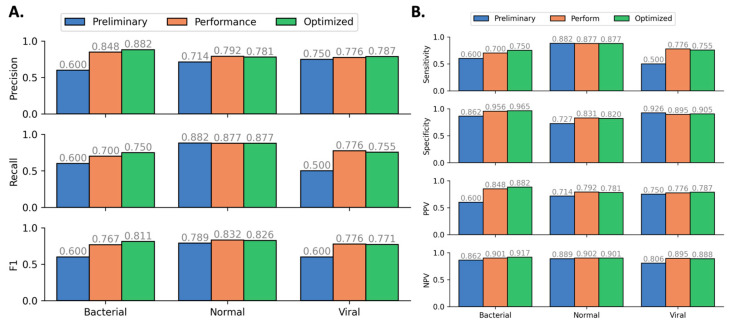
(**A**). ML performance improvement of CatBoost model during ML optimization process. (**B**). Diagnostic performance improvement of CatBoost model during ML optimization process.

To optimize our model selection process for performance, we discontinued the use of lower-performing models (Baseline, Linear, Decision Tree) in favor of more sophisticated, higher-performing models (LightGBM and CatBoost). This decision was made not from a standpoint of computational efficiency, as the latter models are more demanding in terms of computation, but because the enhanced predictive capabilities and significant improvement in results justified the additional computational resources required. Continuing to train and evaluate the less effective models would not be an efficient use of time or resources, given their comparatively limited performance. This selection of models demonstrates mostly comparable performance, with the CatBoost model showing the closest performance agreement with the Ensemble model. As we began to look for increased model performance, in this round of evaluations, we increased the computational effort expended on training each model. Previously, a single CatBoost model was trained and tested. In this stage, we trained multiple CatBoost models with different parameters. Aside from the Ensemble model (which includes the trained CatBoost models), the CatBoost models had the best performance (Figure 3A). This model showed robust improvements in ML performance metrics (Precision, Recall, and F1) for the bacterial class and marginal improvements for the normal and viral classes during the optimization process (Figure 4A).

Along with common machine learning metrics, we performed a statistical analysis on the Optimized CatBoost Model with metrics that are more aligned with traditional diagnostic benchmarks, based on a one vs. all approach to multi-class analysis (Figure 4B). Using a one vs. all approach, the optimized model’s sensitivity and specificity for each individual target class exceeded 0.75, a reasonable target at this stage to demonstrate proof-of-concept feasibility. While the metrics above treat all classifications (i.e., true positives, false positives, true negatives, false negatives) with equal weights, an important consideration moving forward is the determination of an appropriate penalty to apply for certain misclassifications. To demonstrate the practical implications of this, two examples are as follows: (1) a model prediction of bacterial etiology for a true healthy specimen will likely result in an unnecessary antibiotic prescription; (2) a model prediction of normal etiology for either a true bacterial or a true viral specimen will result in a patient not receiving treatment when it may improve their condition. In the future, the model could be biased to consider that the consequences of each type of misclassification are context-specific and not always equal. Making these weights non-uniform across the confusion matrix during training will improve the model’s practical utility.

### 3.3. Feature Importance Downselection

**Figure 5:**
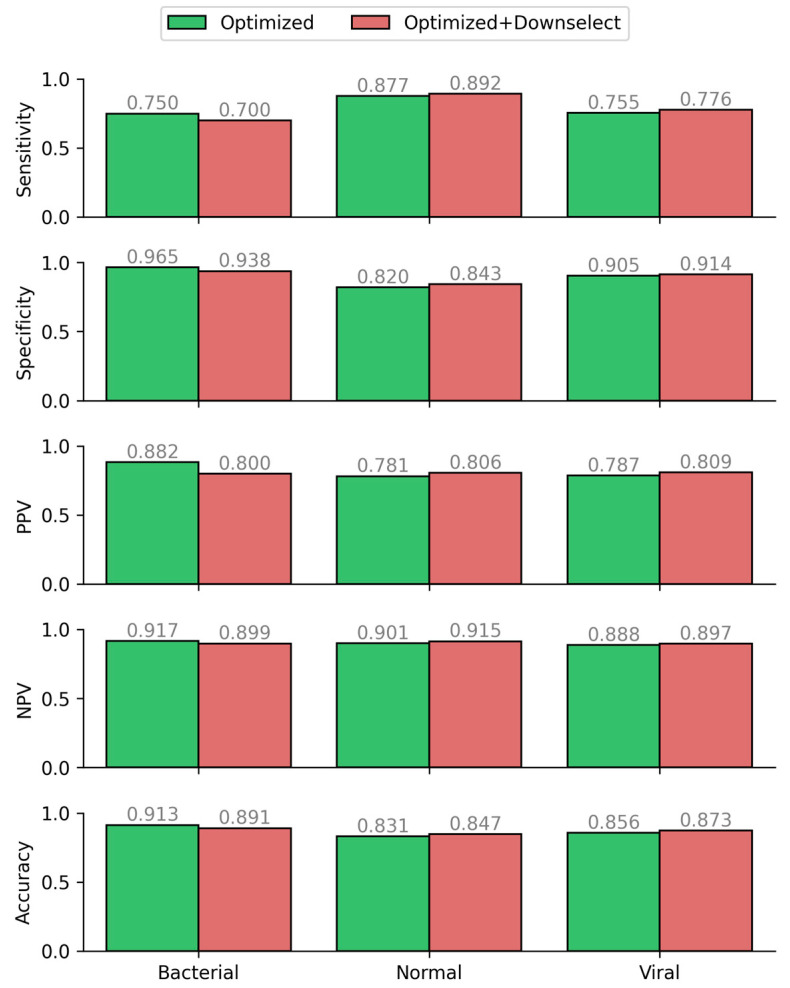
Diagnostic performance metrics of the feature downselected model compared to the base optimized model trained prior to feature downselection.

One of the objectives of this research was to make downselections from the initial list of potential biomarkers to a recommended assay panel, with the expectation that this subset of candidate biomarkers may have implications in future diagnostic algorithms and devices. To this end, correlation ratios with infection identifiers calculated during exploratory data analysis, SHAP feature importance values, and permutation feature importance metrics during explainable model development [45] (Figure 2, SI Figures S3 and S4), would both suggest the same top-8 biomarkers be used in a final panel: CRP, TRAIL, MxA, NGAL, Ferritin, PCT, CXCL10, and TNF-alpha. Downselection to this list of biomarkers requires retraining a new model, as the previously best-performing model from before used data from features that are now removed from consideration. This would be expected to affect model performance: it will remove “noisy” contributions from unhelpful features that have little contribution to model predictions. This newly trained and optimized algorithm (with the downselected set of biomarkers) had a performance nearly identical to the previous model, with slight losses classifying bacterial infections and slight gains classifying viral and normal specimens (Figure 5). With similar performance metrics Figure 3C and Figure 5), the downselected model has the distinct advantage of having fewer features—all things being equal, simpler solutions are usually preferred [46]. Along with easier understandability, there are computational benefits as well as practical advantages for future assay design. On the computational side, models with fewer features require less computing power for training, can be trained more quickly, and are less vulnerable to spurious outputs due to abnormal values in non-important features. On the biomedical and clinical side, fewer biomarker measurements require fewer biochemical assays to be performed in order to evaluate a new specimen—thus reducing the cost and time per sample to be evaluated.

Model performance exhibited a plateau in later development stages, prompting a closer examination of training curves. Figure S5 illustrates the training and testing logloss at each iteration for the optimized and downselected model, which is the highest-performing individual model at this stage. Each “learner” denotes a model trained and tuned on a data subset via cross-fold validation, eventually combined into a single, unified model. Initially, all learners show expected learning behavior from the training dataset, with initially high logloss values decreasing monotonically. However, as training progresses, some curves begin to plateau, indicating a variance in learning capacity and efficiency among learners. Specifically, learners that achieve lower logloss values, nearing 0.05, demonstrate superior training data modeling. A training curve plateau suggests that further iterations yield marginal performance improvements, likely due to models reaching their maximum learning potential given their complexity and the available data. Testing curves for all learners initially show a monotonic decrease for about 30 iterations, reflecting improved model generalization to the testing dataset. The variance in testing curve plateau points underscores differences in learners’ generalization capabilities. This observed variability, both in training and testing performance, underscores the necessity of exploring multiple model configurations in machine learning. Notably, some training curves continue to decrease at a reduced rate, even as their corresponding testing curves plateau, suggesting overfitting. This phenomenon, characterized by a widening gap between testing and training performance at higher iterations, indicates models could be learning training data idiosyncrasies rather than generalizable patterns. The presence of outliers—particularly when shuffled in our relatively small training dataset—could have created testing splits that do not accurately reflect the training data, which might also contribute to the observed gap between the training and testing performance curves. Additionally, some learners are halted earlier than others, a potential indication of Auto-ML detecting performance divergence between testing and training. The observed overfitting and instances of learners reaching their learning capacity, especially given our dataset’s limited size, point to the need for additional training data as a potential solution.

### 3.4. Model Evaluation on a Blinded Sample Set

**Figure 6:**
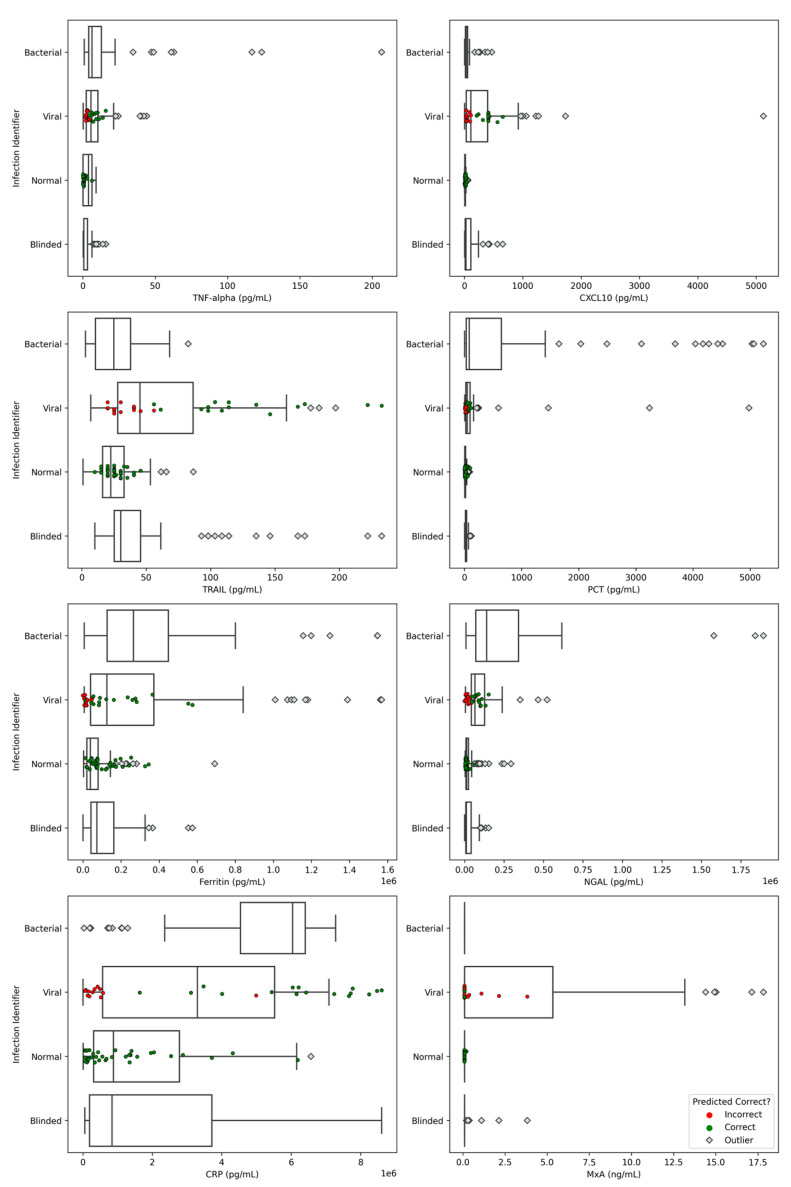
Biomarker distributions obtained from the evaluation of a downselected, optimized model on blinded sample set. Incorrect and correct infection identifier status is overlaid on plotted data points as well as monotonic decrease beyond 1.5× the interquartile range.

To evaluate the optimized model trained on the downselected features, a small set of 65 samples (Table 2) were experimentally analyzed using the same assay panel as that in the data utilized for training, testing, and optimizing, and were evaluated using the new model. None of these samples were used in any of the previous model training and testing iterations described above. The infectious etiology of these specimens, 16 Chikungunya Virus and 49 normal specimens, was blinded to the modeling team. Chikungunya virus, a mosquito-borne enzootic RNA virus that is prevalent in tropical regions, was previously identified in these human sera samples by PCR. The model inference was performed using the optimized model with downselected features, and the model predicted 0 bacterial samples, 37 normal samples, and 28 viral samples. Distributions of the biomarker concentrations from the blinded samples, alongside distributions for each of the classes from the training data, are shown in Figure 6. The individual markers are placed in the class that the ML model classified them to, and they are color-coded by where the algorithm placement was correct. This can be particularly useful in comparing the distributions of the blinded set to the training data, as well as identifying what the model focused on when making classifications. For example, several samples that were incorrectly classified as viral had elevated MxA, which aligned well with the training distribution. Most importantly, the overlapping concentration distributions across the different infection identifiers shown in Figure 6 illustrate the importance of using a multi-biomarker signature, rather than relying on one or two biomarkers to make diagnostic decisions.

**Figure 7:**
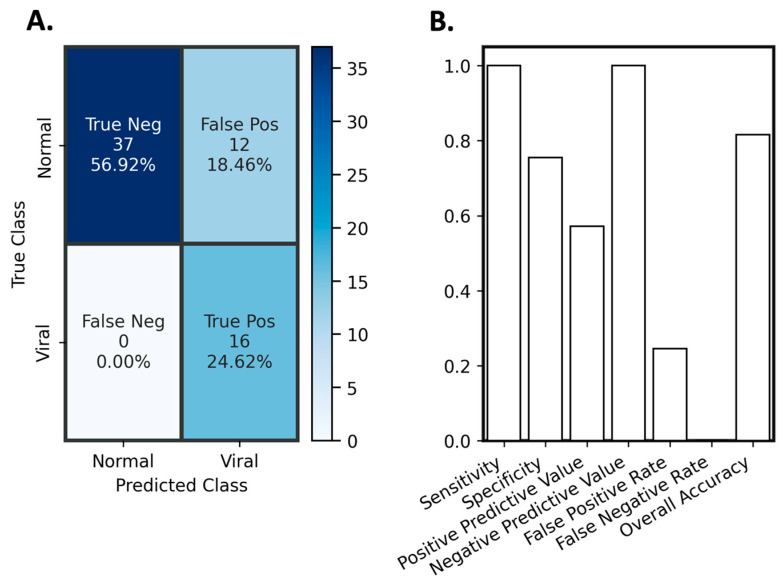
Diagnostic evaluation of optimized classification model with downselected features on a blinded sample set. (**A**) Confusion matrix for blinded evaluation and (**B**) Diagnostic performance of the optimized model on blinded samples.

A confusion matrix for the classes of viral and normal predicted during the blinded evaluation is shown in Figure 7A; since there were no bacterial true positive or false positive predictions, the bacterial class was omitted from the figure. The model correctly identified all 16 true viral infections, with only 12 false positive viral infections and 0 false positive bacterial infections. In this evaluation, the assay had 100% diagnostic sensitivity and 75% specificity. Diagnostic performance metrics are shown in Figure 7B. These results were impressive for a first attempt, especially given the modest size of our training data set (i.e., 221 samples). Furthermore, the blinded dataset only included two classes (i.e., normal and viral), whereas the model was trained to discern between three classes. As the model did not mistakenly predict any samples as belonging to the missing bacterial class, it confirmed that it effectively learned and applied decision boundaries that were established during training. While the source of infection is never known a priori, model performance would be expected to be improved had it only been trained using normal and viral training data. We do note the decrease in specificity from model training to evaluation on this blinded dataset. The higher-than-expected rate of false positives could be attributed to an overlap in biomarker expressions between healthy and viral samples. Certain features of the healthy class may closely resemble those of the viral class, particularly in scenarios that were not encountered or were underrepresented during training. The model’s high sensitivity to biomarker signatures associated with viral infections might have exacerbated these misclassifications.

To further investigate the difference in performance from training to evaluation on the blinded sample set, we compared the distributions of the blinded viral specimens to that of the viral class from the training dataset (SI Figure S6). As noted, all viral specimens from the blinded dataset were from an organism, Chikungunya virus, that was not a part of the training dataset, which contained specimens infected with nine other viral organisms. We found four host biomarkers that had significantly different distributions in Chikungunya virus than in the distributions used for training the classifier: CXCL10, TRAIL, CRP, and MxA. This is likely a substantial contributing factor to the increased false positive rate between training and blinded analysis. Additionally, it highlights the utility of a host biomarker signature over single-plex or even 2- and 3-plex assays, and the importance of training data, particularly in the face of unknown infectious agents.

Further expanding on the difference in viral biomarker distributions, we isolated the individual viruses used in training and compared the biomarker distributions with the Chikungunya virus (from the blinded dataset) in SI Figure S7. MxA was observed to be at the assay’s minimum threshold for six viruses, including MxA. This observation is of note as it would seemingly offer evidence to counter the broad discriminatory utility of MxA to discern viral infections, beyond the causative agents used in the previous literature studies. Other comparisons across biomarker distributions would suggest the potential of certain biomarkers to specifically identify different viral organisms—or at the least, narrow the focus (i.e., PCT to isolate CMV, TNF-alpha to isolate CMV and EBV, and Ferritin to further differentiate CMV from EBV). While individual sample sizes of the causative organisms are too low for statistically meaningful comparisons, the differences across biomarker distributions merit future investigation.

## 4. Conclusions

This manuscript describes a machine learning-based classification approach for discriminating between three disease etiology classes: bacterial, viral, and normal using host-based soluble protein biomarkers, including cytokines and acute-phase proteins, for which commercial off-the-shelf immunoassays already exist. To this end, we collected protein biomarker data from 221 human sera samples of known disease etiology and used this to train a series of increasingly optimized models through feature downselection and model parameter tuning, ultimately resulting in an optimized classifier against our three infection classes.

Training a machine learning model has historically required significant expertise and computational resources. In this work, the number of features and the overall volume of the training dataset were relatively modest and model training could be accomplished on a consumer-grade machine. With on-demand computing power now readily accessible and inexpensive, even larger scale datasets can now be readily analyzed without dedicated resources. The optimized model with downselected features described in this manuscript is lightweight at only 471 KB on disk. In its current implementation, it is stored as a Python binary file that can be loaded using any compatible Python interpreter. After bundling all model dependency packages and the script necessary to load and execute model inference, the total software package size is 271 MB without any compression. While this could likely be reduced, the package could readily be deployed in its current form to several cloud or mobile device architectures.

Auto-ML was leveraged in the workflow presented herein to rapidly evaluate different model architectures. This approach removes the burden of a priori selection of model type, which would require deep domain expertise to understand model strengths and weaknesses. Rather, Auto-ML rapidly iterates through multiple algorithms and proceeds to optimize the hyperparameters of only the most promising models. This allows critical decisions on model architecture and hyperparameter values to be automatically decided based on performance, and not operator expertise. In this sense, the approaches described in this work could be readily implemented in other life sciences applications. While we selected a particular library for use in this work, other auto-ML libraries (e.g., Auto-sklearn, H2O autoML, TPOT, AutoGluon) exist and have relative strengths and weaknesses that must be evaluated in the context of any particular application [47]. In applying this Auto-ML approach to identifying a bacterial vs. viral biomarker classifier, we identified a Categorical Boosting (CatBoost) model. This model is itself a gradient-boosting technique meant to optimize classification models from an ensemble of weaker models as the best approach. We eventually arrived at a downselected model with sensitivity/specificity values of 0.7/0.938 for the bacterial class, 0.776/0.914 for the viral class, and 0.892/0.843 for the normal class. Two widely used agnostic infectious diagnostics for the identification of bacterial etiology in clinical use are CRP and PCT. In practice, these diagnostics give widely varying degrees of diagnostic performance when not used in combination with other clinical measures like white blood cell count, etc. A recent systemic review and meta-analysis of the literature for bacterial infections reported average sensitivity/specificity of 0.88/0.81 and 0.75/0.67 for PCT and CRP, respectively [48]. Overall, then, our initial effort fared very well, especially considering it tackled a more difficult scenario of three-class differentiation instead of the two-class situation described above. This model was then evaluated on an entirely blinded dataset consisting of two sample classes (viral and naïve), in which it had 100% diagnostic sensitivity and 75% specificity, impressive outcomes given the modest size of our training data set. From a future assay optimization standpoint, detailed analysis of both our training and blinded evaluation datasets reveals apparent patterns in the host protein biomarker distributions, illustrating the need for a biomarker signature to be used in the classification of infectious etiology, rather than a single biomarker. The preliminary assessment of our model performance is encouraging as the apparent gap we saw in the logloss performance of the training and test curves for our optimized models (SI Figure S5) suggests that their performance could be further improved in future iterations, likely with additional training on larger sample sets. Furthermore, while the AutoML library employed in this study did implement scaling methods to diminish the impact of outliers, these methods were not extensively explored beyond the default settings for each model type. This could also be a possible explanation, combined with the natural resilience of tree-based ensemble models like CatBoost and XGBoost to outliers, for their consistently superior performance throughout the various stages of model development. Consequently, it is conceivable that exploring alternative scaling techniques could enhance the performance of other model architectures by more effectively mitigating outlier effects. Finally, while not pursued further, the number of biomarkers present in future iterations of this bacterial/viral classifier could possibly be decreased even further with limited deterioration in diagnostic performance. As noted earlier, in consideration of SHAP feature importance scores, ferritin and TNF-alpha are two biomarkers that would be likely candidates for future removal.

While not explicitly considered in this work, it is important to note that in multi-class problems, different classes may have different levels of importance or relevance, and choosing appropriate performance metrics depends on the specific context and objectives of the problem. Sometimes, other metrics such as macro or micro-averaging may also be used to calculate aggregate performance measures across multiple classes. Within a diagnostic context, both false positives and false negatives carry risk. However, the precise weight of those risks can vary widely depending on the diagnostic application scenario, such as the differences between cancer diagnosis versus infectious disease diagnosis. Infectious disease diagnostics, especially those at the point of care, penalize false negatives more as they can impact not only clinical decisions related to treatment but also prevention/quarantine measures that may impact community spread, as has been seen for SARS-CoV-2 management [49].

In conclusion, we have identified a flexible approach for applying machine learning-based classification tools to identify human host biomarker signatures for infectious disease diagnostic applications. In this instance, we applied this classification scheme to identify a multiplexed protein biomarker signature that had promising initial diagnostic performance against a blinded sample set for viral/bacterial/normal diagnosis. We have identified areas for improvement in future modeling efforts, including refining feature engineering and scaling techniques to enhance the distinction between healthy and viral classes, especially in the presence of novel pathogens and varied class distributions, and conducting extended validation and robustness testing with a broader array of external datasets that expose the model to diverse class distributions, co-infections, and novel pathogens. Training this model with larger, well-curated human infectious disease sample sets would undoubtedly strengthen the model and improve performance. While the test case chosen for this effort was agnostic bacterial/viral disease classification, this Auto-ML method could easily be applied to any scenario where a disease diagnostic based on multiparametric host biomarker interpretation is required. Once designed and validated, these host-centered diagnostics can inform clinical decision making by providing an understanding of patient’s past infection status, their current immune profile, infection severity, and disease prognosis. In this regard, they can be seen as complementary to pathogen-focused diagnostics and can help address existing gaps in the global ability to respond to novel or unidentified threats.

## Figures and Tables

**Table 1:** Summary of clinical samples used for model classifier training.

Infectious Agent	Number of Samples	Classifier
*Escherichia coli*	14	Bacterial
*Enterococcus faecalis*	7	Bacterial
*Enterobacter asburiae*	4	Bacterial
*Citrobacter koseri*	3	Bacterial
*Entercoccus* sp.	3	Bacterial
*Klebsiella pneumonaie*	3	Bacterial
*Staphylococcus* sp. *(coag neg)*	3	Bacterial
*Staphylococcus aureus*	3	Bacterial
*Borrelia burgdorferi*	6	Bacterial
*Chlamydia trachomatis* and *Neisseria gonorrhoeae*	1	Bacterial
*Chlamydia trachomatis*	3	Bacterial
*Neisseria gonorrhoeae*	4	Bacterial
*Treponema pallidum*	5	Bacterial
*Enterococcus faecalis*, *Pantoea agglomerans*, *Kleb. Pneumo*	3	Bacterial
Cytomegalovirus	6	Viral
Epstein–Barr Virus	14	Viral
Hepatitis A	2	Viral
Hepatitis B	13	Viral
Hepatitis C	17	Viral
Influenza A	7	Viral
Influenza B	1	Viral
Influenza sp.	1	Viral
Dengue virus	10	Viral
Naive Sera	88	Normal

**Table 2:** Summary of clinical samples used for blinded evaluation.

Infectious Agent	Number of Samples	Classifier
Chikungunya Virus	16	Viral
Naive Sera	49	Normal

## Data Availability

All data presented in this study are available within the article and Supplementary Materials.

## Supplementary Materials

The following supporting information can be downloaded at: https://www.mdpi.com/article/10.3390/diagnostics14121290/s1, Figure S1. Individual biomarker distributions for all protein analytes in this study; Figure S2. Exploratory analysis of training dataset outliers, calculated as outside 1.5 times the interquartile range of an individual biomarker, for a given infection identifier (as opposed to an individual biomarker across all infection identifiers) in the downselected biomarkers of the training dataset; Figure S3. Additional preliminary modeling metrics; Figure S4. Feature importance during modeling; Figure S5. Training curves on optimized, downselected model; Figure S6. Statistical analysis comparing the virus specimens from the blinded testing (all Chikungunya virus) to the virus specimens used in model training (which contained no Chikungunya specimens); Figure S7. Biomarker distributions comparing the viral causative organisms used in the model training, in addition to the distribu-tion of Chikungunya virus (CHIKV) used in blinded testing.
