# Supplementary material for: Application of a Machine Learning-Based Classification Approach for Developing Host Protein Diagnostic Models for Infectious Disease
Source: Diagnostics (Basel). 2024 Jun 18;14(12):1290. doi: 10.3390/diagnostics14121290 (PMC11202442; doi:10.3390/diagnostics14121290)
Supplement: Supplementary file 1 [file diagnostics-14-01290-s001.zip › diagnostics-3026410-supplementary.pdf]

diagnostics

MDPI

Supplemental Figures

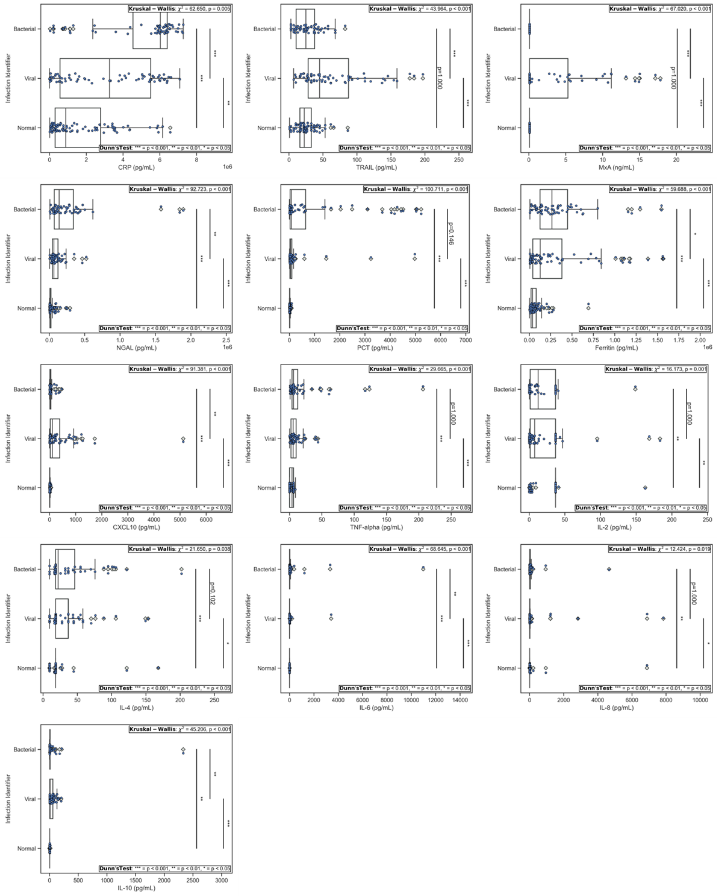

SI Figure S1. Individual biomarker distributions for all protein analytes in this study. Each biomarker concentration for each sample is shown as an individual circle with small jitter applied for visualization, while outliers of the distribution (>1.5x the interquartile range) are shown as diamonds. The Kruskal-Wallis chi-squared value and p-value are shown as an inset in the top right of each sub-figure; significance in pairwise comparisons made using Dunn's Test are indicated with p < 0.05 (*), p < 0.01 (**), and p < 0.001 (***).

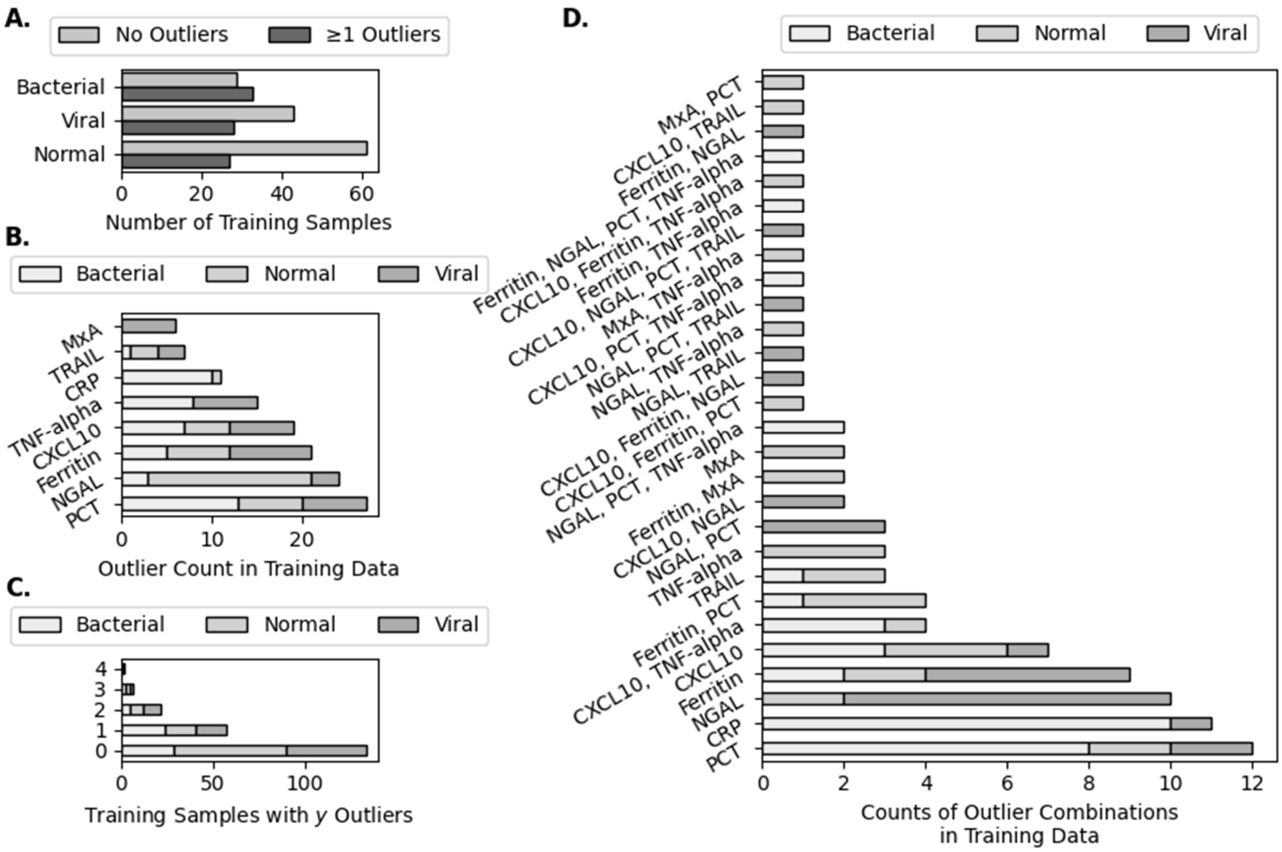

SI Figure S2. Exploratory analysis of training dataset outliers, calculated as outside 1.5 times the interquartile range of an individual biomarker, for a given infection identifier (as opposed to an individual biomarker across *all* infection identifiers) in the downselected biomarkers of the training dataset. **A.** The incidence of one or more outliers in a single training sample, across the three infection identifiers. Bacterial infections had the highest count and percentage of samples that contained at least one outlier; normal sera had the lowest count and percentage of samples that contained at least one outlier. **B.** The number of times each of the downselected biomarkers were found to be an outlier, stacked by infection identifier. PCT had the most outliers, and the majority of those occurred in a bacterial specimen. Conversely, MxA had the fewest outliers; when outliers did occur, they were always in viral specimens. In healthy specimens, NGAL had the most outliers. **C.** The occurrence of multiple outliers across infection identifiers. There were 7 specimens that had 3 outlier biomarkers (3 bacterial, 2 normal, and 2 viral); there were 2 specimens that had 4 outlier biomarkers (1 bacterial, 1 normal). **D.** The space of combinations of outliers present in any individual sample, separated by infection identifier. While the top 5 counts of any outlier combinations were actually individual biomarkers, there were combinations of outlier biomarkers that were more frequent than individual biomarker outliers.

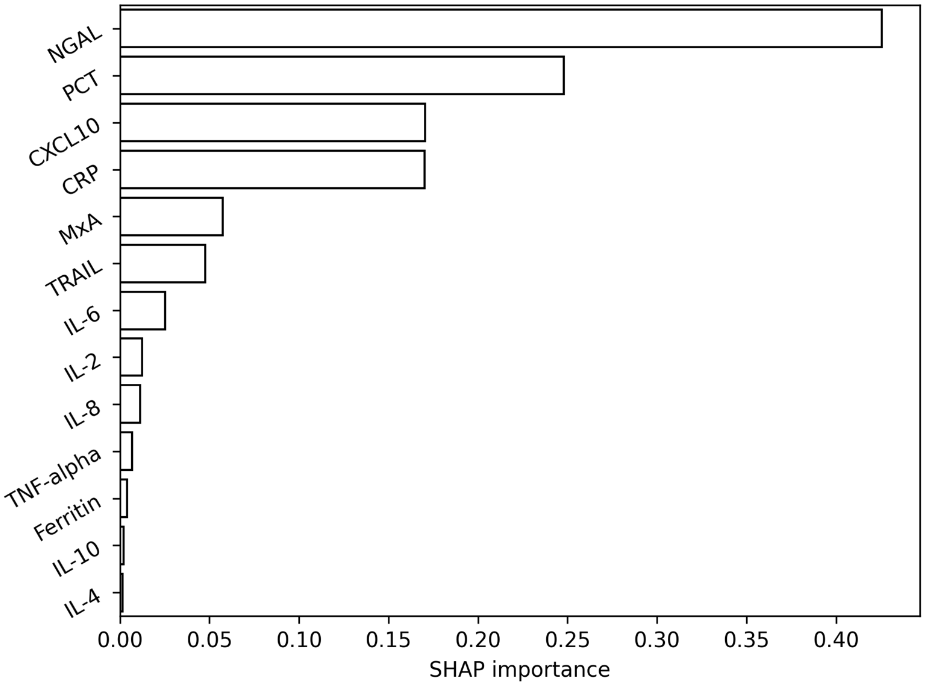

SI Figure S3. Additional preliminary modeling metrics. SHAP feature importance values for the Decision Tree model during preliminary modeling show general alignment on the most important features with the Infection Identifier correlation ratios. Importantly, there is mostly agreement with the Infection Identifier correlation ratios between the least important features, which aids in downselection of biomarkers IL-8, IL-10, and IL-4 from future model consideration.

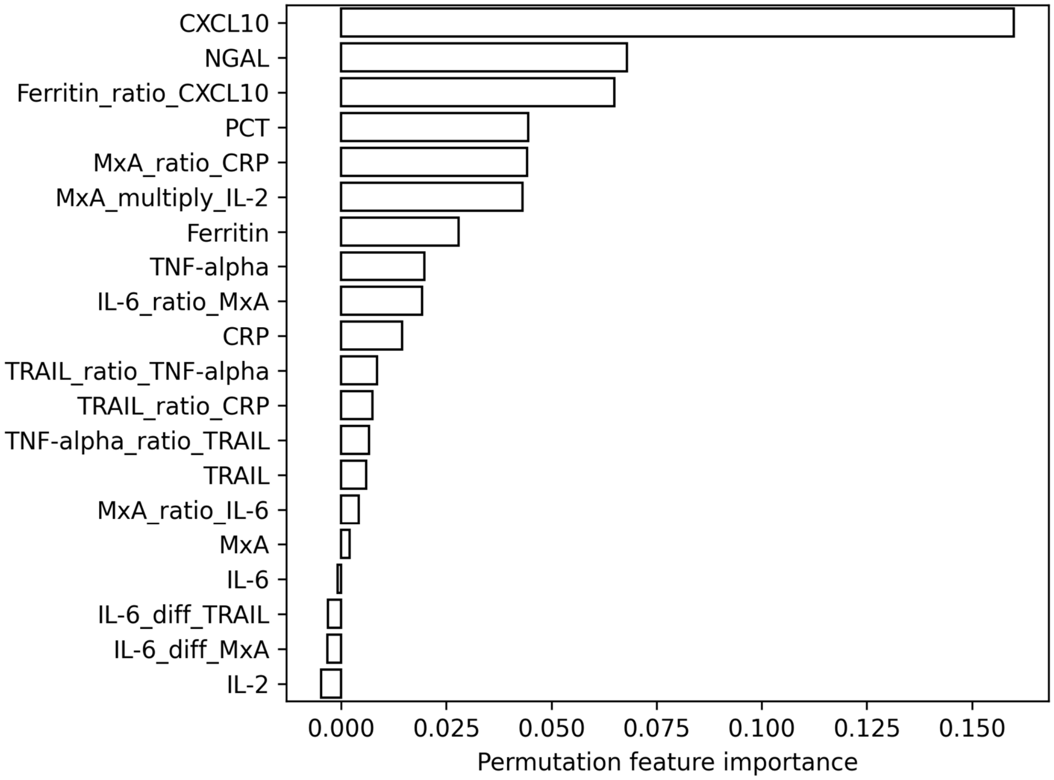

SI Figure S4. Feature importance during modeling. Permutation feature importance for CatBoost with Golden Features during performance modeling, used in part to select final biomarker panel (CRP, MxA, NGAL, Ferritin, Procalcitonin, CXCL10, and TNF-alpha).

Golden Features models evaluate combinations of individual features may have improved predictive power. Nomenclature for Golden Features follows the following syntax: the first feature, underscore, an operator (i.e., "ratio" for division, "multiply", "diff" for subtraction), underscore, and the second feature. Features without this syntax are the standalone features with no modification.

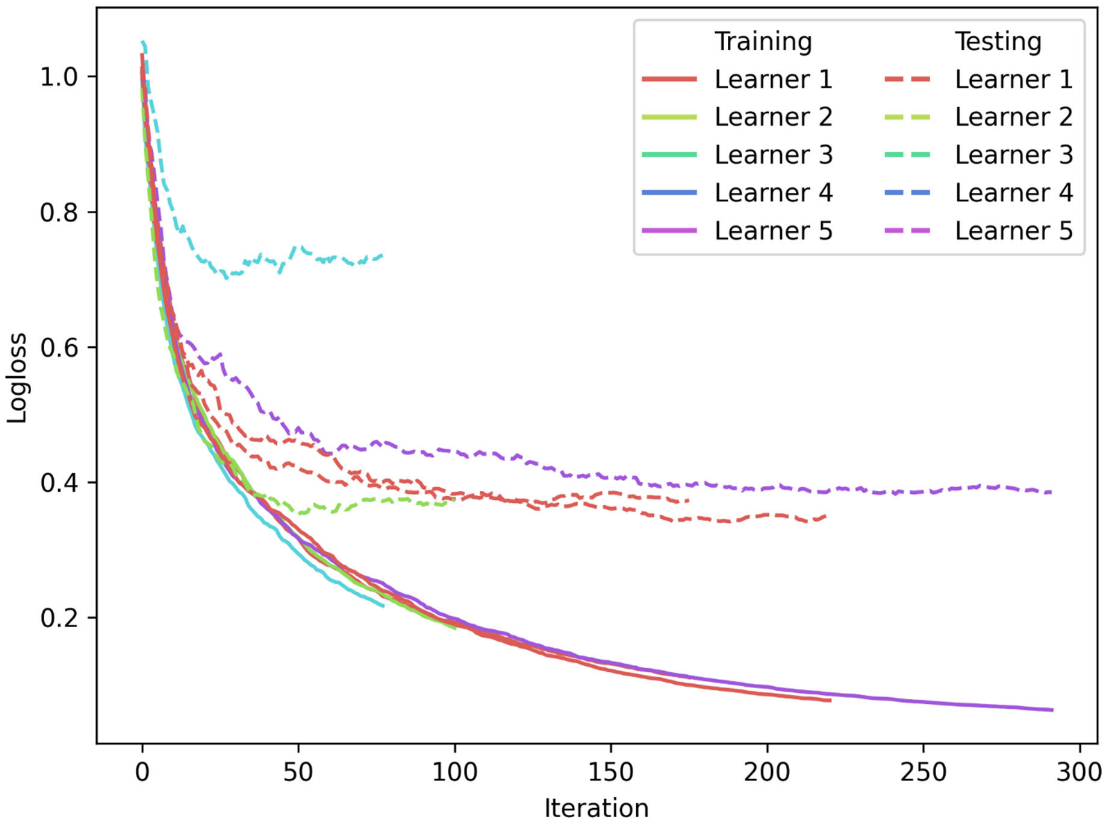

SI Figure S5. Training curves on optimized, downselected model. These logloss curves, representing the micro-averaged logloss across all classes, demonstrate that the model is learning from the training data but does reach its learning capacity and shows some evidence of overfitting. Increased training data set volume (more clinical samples) would be expected to mitigate this and further improve model performance.

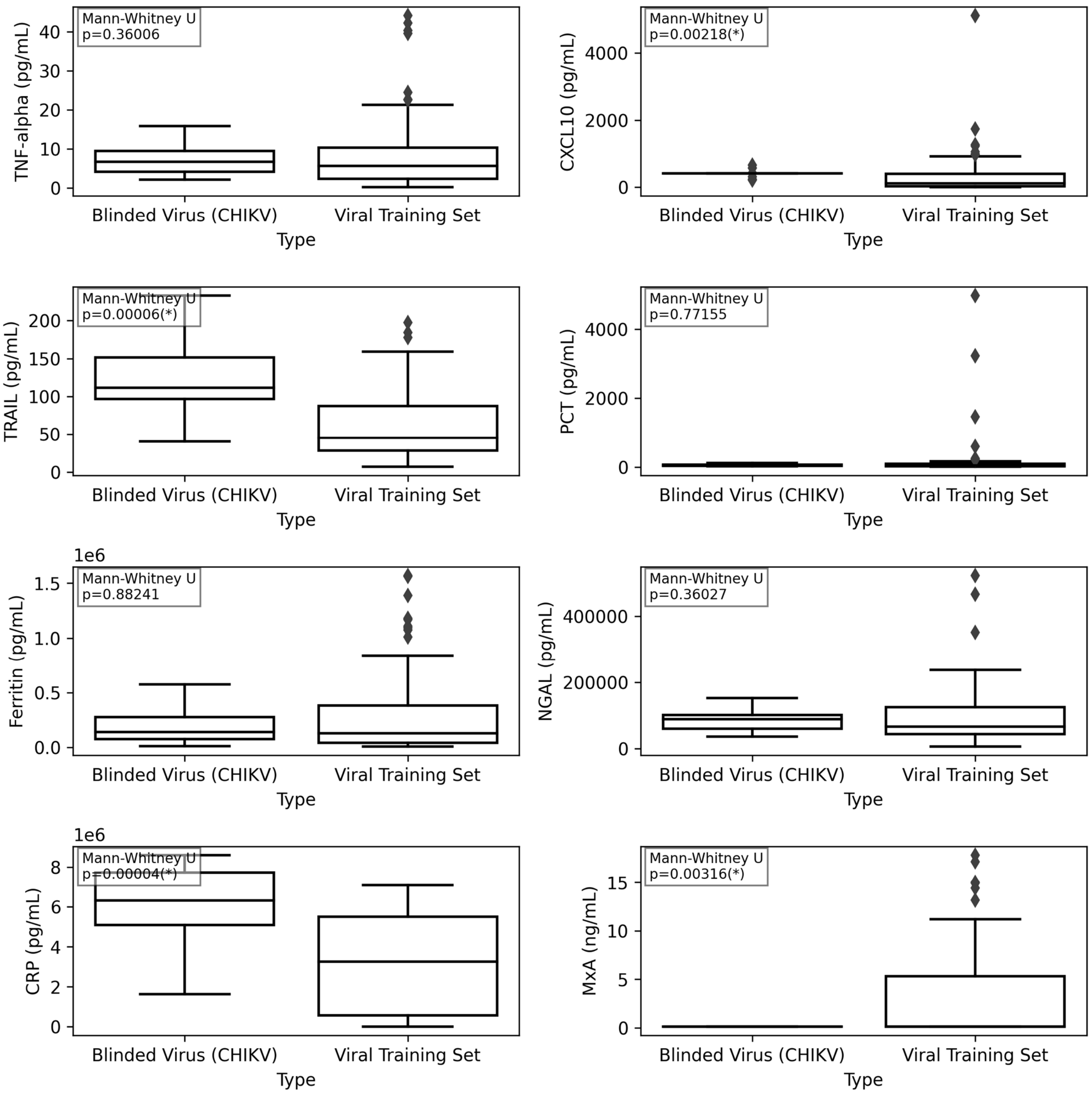

SI Figure S6. Statistical analysis comparing the virus specimens from the blinded testing (all Chikungunya virus) to the virus specimens used in model training (which contained no Chikungunya specimens). Pairwise statistically significant differences were found in the distributions of CXCL10, TRAIL, CRP, and MxA. This may have contributed to the increased false positive rate noted during blinded validation.

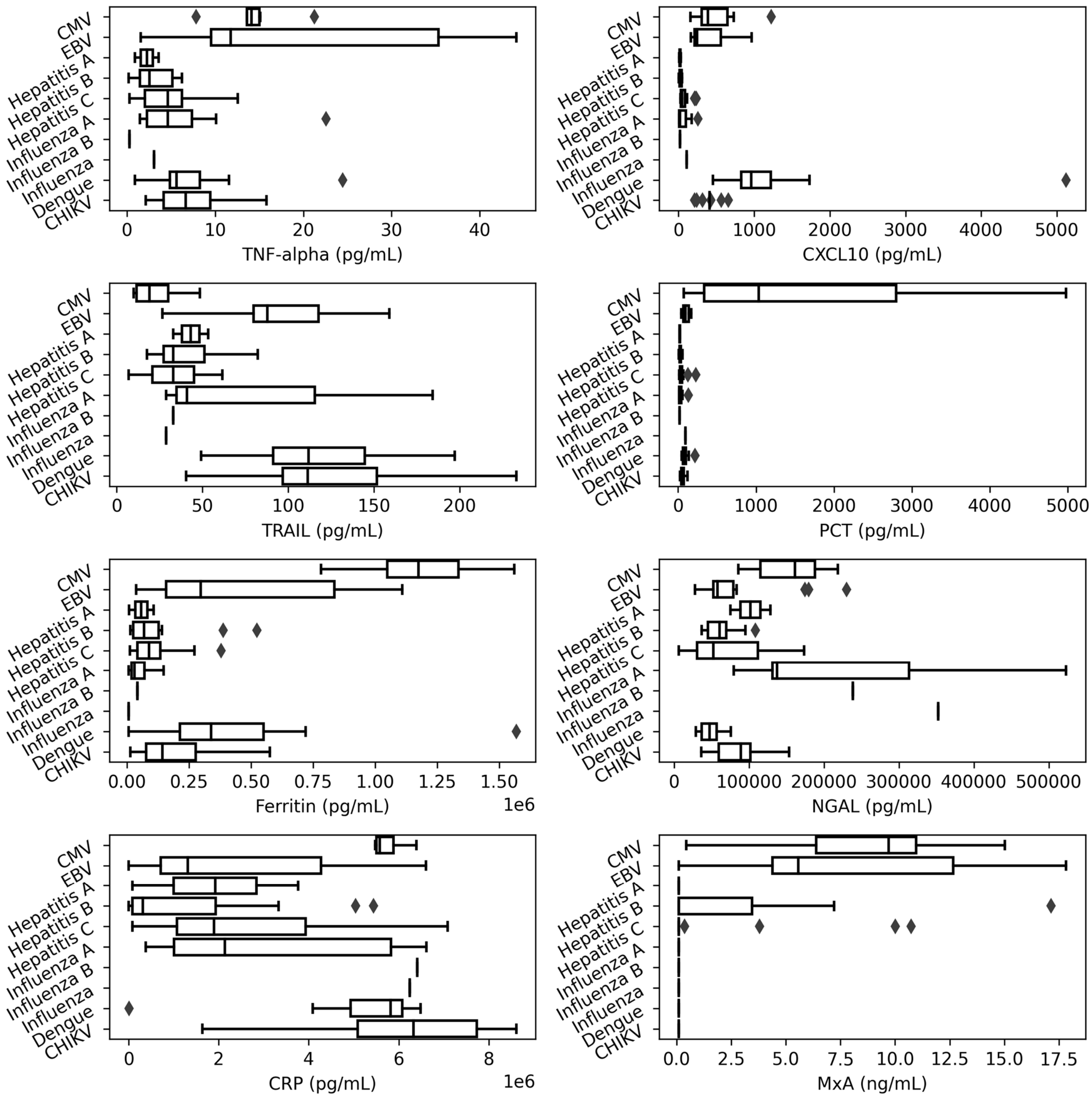

SI Figure S7. Biomarker distributions comparing the viral causative organisms used in the model training, in addition to the distribution of Chikungunya virus (CHIKV) used in blinded testing. This expands on SI Figure 6, by separating the individual viral organisms from the parent class. These distributions demonstrate the necessity of multiple biomarkers as features, in addition to the importance of a dataset with a diverse set of infectious agents – even within a particular infection identifier set. The sample size for some organisms was very low, limiting a full set of meaningful pairwise statistical comparisons, there are interesting trends for certain biomarkers that merit future investigation. While caution must be exercised due to the small volume of samples, there may be potential use in machine learning models that can utilize host biomarker signatures to identify specific infectious viruses. Such a model would have broad utility, including the selection of appropriate therapeutics, monitoring treatment response, and determining the efficacy of prophylactic countermeasures.
